# *Cryptosporidiosis* and *Isosporiasis* among HIV-positive individuals in south Ethiopia: a cross sectional study

**DOI:** 10.1186/1471-2334-14-100

**Published:** 2014-02-22

**Authors:** Mekonnen Girma, Wondu Teshome, Beyene Petros, Tekola Endeshaw

**Affiliations:** 1 Microbiology-Parasitology Unit, School of Medicine, Hawassa University, Hawassa, Ethiopia; 2 School of Public and Environmental Health, Hawassa University, Hawassa, Ethiopia; 3 Department of Biology, Addis Ababa University, Addis Ababa, Ethiopia; 4 The Carter Centre, Addis Ababa, Ethiopia

**Keywords:** *Cryptosporidium spp*, *I. belli*, Ethiopia, HIV

## Abstract

**Background:**

*Cryptosporidium spp* and *I. belli* are intestinal opportunistic infections associated with HIV/AIDS. A decline in the incidence of these opportunistic infections due to HAART was reported. We aim to investigate these parasites among HAART naïve and experienced HIV patients in south Ethiopia.

**Methods:**

A cross sectional study was carried out among 268 HIV- positive patients between January and September, 2007. Interview with questionnaires and document reviews were used to collect data. Stool samples were obtained from each patient and parasites were examined by direct, formol-ether and modified Ziehl-Neelsen stain for *Cryptosporidium spp* and *I. belli*. Univariate and multivariate analysis were carried out. Level of significance was set at p-value of 0.05.

**Results:**

A total of 268 patients participated in the study. The mean age was 34.0 (±1 SD of 8.34) years. Females constituted 53.4% (143) of the study participants. Half of the study participants were on HAART; majorities (85.8%) of such patients were within the first year of treatment. The prevalence of *Cryptosporidium spp* was 34.3% (92/268) and *I. belli* was 1.5% (4/268). Dual infection was detected in two patients (0.75%). The crude analysis revealed significant reduction in the odds of *Cryptosporidium spp* infection among patients who have started HAART (crude OR = 0.59, 95% CI 0.35, 0.98). The adjusted analysis remained in the same direction but has lost significance (Adj OR 0.65, 95%CI 0.35, 1.24). No differences in the risk of developing infection with *Cryptosporidium spp* were observed between groups based on most recent CD4 counts, sex, duration on HAART and age (p > 0.05 for all variables). Patients with *Cryptosporidium spp* were more likely to report vomiting [Adj OR 2.34 (95% CI 1.22, 5.41)], weight loss [Adj OR 2.10 (95% CI 1.15, 3.81)] and chronic diarrhea [Adj OR 3.35 (95%CI 1.05, 10.63)].

**Conclusion:**

There is high burden of infection with *Cryptosporidium spp* among HIV infected individuals in southern Ethiopia but that of *I. belli* is low. We recommend considering infection with *Cryptosporidium spp* in HIV infected people with chronic diarrhea, weight loss and vomiting for HAART naïve patients and/or for patients who are within the first year of starting HAART.

## Background

HIV infected patients are susceptible to a variety of common and opportunistic infections due to progressive decline in their immunity status. Intestinal protozoan parasites that cause a mild or self-limited disease in immunocompetent individuals can cause protracted and severe diarrhea in immunocompromised patients. Among the intestinal protozoa that cause intestinal infections in immunocompromised patients, *Cryptosporidium parvum* and *I. belli* are most frequently encountered [1,2].

Highly active antiretroviral therapy (HAART) can restore immunity by increasing memory and naïve CD4^+^ cells against pathogens [3,4]. In the absence of a vaccine and affordable HAART, people living with HIV/AIDS, especially in developing countries, remain at risk of opportunistic infections (OIs) [5].

In Ethiopia, some studies have been conducted on the distribution and prevalence of opportunistic parasites among HIV infected individuals [5,6]. The high rates of occurrence of *Cryptosporidium parvum* and *Isospora belli* in Ethiopia indicates that both are major public health concern specifically among HIV infected patients. The experiences of developed nations have proven that HAART reduces disease burden and improves the wellbeing and productivity of AIDS patients. Thus, the aim of this study was to assess the prevalence of intestinal opportunistic infections among HIV infected patients in south Ethiopia.

## Methods

### Study setting

The study was conducted in Yirgalem hospital which is located at a distance of 347 km south of Addis Ababa, the capital city of Ethiopia.

### The study design and population

This was a cross sectional study which was conducted over a period of January - September, 2007. A total of 268 HIV infected patients who were on chronic care follow up at Yirgalem Hospital’s HIV clinic were sampled consecutively.

Sociodemographic characteristics and symptom related information were collected by directly interviewing patients using a structured questionnaire. The duration on treatment and types of HAART drug regimen and other information including the most recent CD4 cells counts were obtained from HIV care clinic registers (esp. ART register) and appropriate formats including the HIV care follow up forms and patient intake forms. Patient follow up forms were first used to retrieve the recent CD4 count and for how long the patient has taken HAART. We have used the ART register in cases where we were not able to get either of the information from the follow up form.

Diarrhea in this paper is defined as a subjective report by the study participants as having passage of unformed stool for more than 2 or 3 times a day. Moreover, we used reported duration of one month cut off to differentiate acute from chronic diarrhea.

### Stool collection and processing

Single stool samples were collected in a leak proof vial and subjected to microscopy. SAF (Sodium acetate-Acetic acid-Formalin) preserved specimens and air dried smear were taken to the EHNRI’s (Ethiopian Health and Nutrition Research Institute) parasitology department for analysis. Each specimen was examined by direct wet-saline, formol-ether concentration and also stained by modified Ziehl-Neelsen/MZN to detect the oocysts. The EHNRI’s operating procedure for identification of coccida parasites was used. The size of the oocysts was utilized to differentiate *Cryptosporidium spp* from *Cyclospora* oocysts. A sample is labeled as positive for *Cryptosporidium spp* if the oocysts’ size ranges between 4–6 μm.

### Ethical consideration

The ethical committee of Addis Ababa University (AAU), Biology Department approved the study proposal. Written informed consent was obtained from each study participant.

### Data analysis

The data were entered, cleaned and analyzed using SPSS, version 16.0. Univariate analysis was first run to detect for factors that are associated with *Cryptosporidium spp* infections. Then adjusted analysis was performed by entering variables that have shown significance during univariate analysis or have been previously reported to be significant in other literatures. For all statistical decisions, the level of significance was set at α of 0.05.

## Result

### Characteristics of study participants

A total of 264 HIV infected patients participated in the study. The overall mean age of the study participants was 34.0 (±1 SD of 8.34). Females constituted 53.4% (143) of the study participants. Half of the study participants have been initiated on HAART. The commonest HAART regimen being used by the study participants was D4T/3TC/NVP, accounting for 63.4% (85/134) out of those patients initiated on HAART. Among patients who were initiated on HAART, 63.4% (85/134) have taken the therapy for more than 7 months and 85.8% (115/134) of such patients were within the first year of treatment (Table 1). *Cryptosporidium spp* infection was detected in 34.3% (92/268) of the study participants where as *I. belli* was identified only in 4 (1.5%) cases. Dual infection was detected in 2 patients (0.75%).

**Table 1:** Sociodemographic, treatment and immunity characteristics of study participants

**Variables**	**Cryptosporidium spp**		**Isospora belli**	
	**Yes**	**No**	**Yes**	**No**
***Age (mean, ±1SD) (n = 268)***	33.5, ±8.1	34.3, ±8.5	35.5, ±5.5	34.0, ±8.3
***CD4 count (n = 268)***				
**≤** ***250***	67 (35.8)	120 (64.2)	3 (1.6)	184 (98.4)
*251-350*	14 (29.8)	33 (70.2)	1 (2.1)	46 (97.9)
**≥** ***351***	11 (32.4)	23 (67.6)	0 (0.0)	34 (100.0)
***Sex (male: female) (n = 268)***	0.74:1	0.96:1	1:01	0.870;1
***Treatment status (n = 268)***				
*On HAART*	38 (28.4)	96 (71.6)	1 (0.8)	133 (99.2)
*Not on HAART*	54 (40.3)	80 (59.7)	3 (2.2)	131 (97.8)
***HAART regimen (n = 134)***				
*D4T/3TC/NVP*	20 (23.5)	65 (76.5)	1 (1.2)	84 (98.8)
*D4T/3TC/EFV*	2 (28.6)	5 (71.4)	0 (0.0)	7 (100.0)
*AZT/3TC/NVP*	15 (41.7)	21 (58.3)	0 (0.0)	36 (100.0)
*AZT/3TC/EFV*	1 (16.7)	5 (83.3)	0 (0.0)	6 (100.0)
***Duration on HAART*** (in months) ***(n = 134)***				
***(n = 134, mean, ± 1SD)***	***8.9, ± 4.0***	***10.1 ± 5.9***	***9.4 ± 5.4***	4.0*
*2-7 months*	14 (28.6)	35 (71.4)		
*> = 8 months*	24 (28.2)	61 (71.8)		

* Among patients who have started HAART, only 1 patient was diagnosed with Isospora belli and no SD was calculated.

### Clinical characteristics

Majority of the patients [90.3% (242)] had complaints of diarrhea. Among patients complaining of diarrhea almost half [48.5% (130)] of them had chronic diarrhea. The next major complaint was abdominal pain accounting for 70.1% (188) of the study participants (Table 2).

**Table 2:** Clinical characteristics of the study participants

**Variables**		**Cryptosporidium spp**		**Isospora belli**	
		**Yes (%)**	**No (%)**	**Yes (%)**	**No (%)**
***Abdominal pain***	Yes	67 (35.6)	121 (64.4)	4 (2.1)	184 (97.9)
	No	25 (31.3)	55 (68.7)	0 (0.0)	184 (100.0)
***Vomiting***	Yes	31 (54.4)	26 (45.6)	1 (1.8)	56 (98.2)
	No	61 (28.9)	150 (71.1)	3 (1.4)	208 (98.6)
***Nausea***	Yes	36 (38.3)	58 (61.7)	1 (1.1)	93 (98.9)
	No	56 (32.2)	118 (67.8)	3 (1.7)	171 (98.3)
***Reported weight loss***	Yes	43 (50.6)	42 (49.4)	2 (2.4)	83 (97.6)
	No	49 (26.8)	134 (73.2)	2 (1.1)	181 (98.9)
***Diarrhea***	No diarrhea	4 (15.4)	22 (84.6)	0 (0.0)	26 (100.0)
	Acute diarrhea	36 (32.1)	76 (67.9)	0 (0.0)	112 (100.0)
	Chronic diarrhea	52 (40.0)	78 (60.0)	4 (3.1)	126 (96.69)
***Total (same for all categories)***		***92 (34.3)***	***176 (65.7)***	***4 (1.5)***	***264 (98.5)***

### Factors associated with Cryptosporidium spp infection

No differences in the risk of developing infection with *Cryptosporidium spp* were observed between groups based on most recent CD4 counts, sex of the study participants, duration on HAART and age category of the study participants (p > 0.05 for all variables). The crude analysis has shown a significant reduction in the odds of *Cryptosporidium spp* infection among patients who have started HAART than among those who have not started treatment with HAART. The adjusted analysis remained in the same direction but has failed to attain level of statistical significance (Table 3).

**Table 3:** **Association between sex, age, CD4 counts and treatment status with** ***Cryptosporidium spp***

**Variables**	**Cryptosporidium spp**	
	**Crude OR (95%CI)**	**Adj OR (95% CI)**
***CD4 count***		
<= 100	1.00	1.00
101-350	0.64 (0.35, 1.15)	0.69 (0.37, 1.29)
>350	0.66 (0.27, 1.57)	0.82 (0.31, 2.16)
***HAART***		
Yes	0.59 (0.35, 0.98)*	0.65 (0.35, 1.24)
No	1.00	1.00
***Sex***		
Male	0.77 (0.46, 1.28)	0.80 (0.47, 1.35)
Female	1.00	1.00
***Duration on HAART***		
No HAART	1.00	1.00
≤ 7 months	0.59 (0.29, 1.20)	0.62 (0.29, 1.29)
> 7 months	0.58 (0.32, 1.05)	0.65 (0.35, 1.24)
***Age, years***		
< 35	1.00	1.00
> = 35	0.72 (0.43, 1.19)	0.76 (0.45, 1.28)

OR = Odds Ratio, Adj = Adjusted, CI = Confidence Interval, HAART = Highly Active Anti-Retroviral Therapy, spp = species, * Statistically significant.

### Clinical symptoms associated with Cryptosporidium spp infection

Next we used logistic regression analysis for clinical symptoms reported by patients to verify their association with *Cryptosporidium spp* infection. Accordingly, patients with *Cryptosporidium spp* were more likely to report vomiting [Adj OR 2.34 (95% CI 1.22, 5.41)], weight loss [Adj OR 2.10 (95% CI 1.15, 3.81)] and chronic diarrhea when compared with patients who didn’t report diarrhea [Adj OR 3.35 (95%CI 1.05, 10.63)] (Table 4).

**Table 4:** **Association between clinical symptoms and** ***Cryptosporidium spp***

**Reported symptoms**		**Cryptosporidium spp**	
		**Crude OR (95%CI)**	**Adj OR (95% CI)**
***Abdominal pain***	Yes	1.22 (0.70, 2.13)	1.11 (0.59, 2.06)
	No	1.00	1.00
***Vomiting***	Yes	2.93 (1.61, 5.34)	2.34 (1.22, 5.41)*
	No	1.00	1.00
***Nausea***	Yes	1.31 (0.78, 2.21)	1.15 (0.64, 2.06)
	No	1.00	1.00
***Reported weight loss***	Yes	2.80 (1.64, 4.79)	2.10 (1.15, 3.81)*
	No	1.00	1.00
***Diarrhea***	No diarrhea	1.00	1.00
	Acute diarrhea	2.61 (0.84, 8.12)	2.22 (0.68, 7.27)
	Chronic diarrhea	3.67 (1.19, 11.26)	3.35 (1.05, 10.63)*

Note: Also adjusted for treatment status (HAART vs. no HAART), * statistically significant.

## Discussion

In this paper it was found that there is high burden of infection with *Cryptosporidium spp* in south Ethiopia with a prevalence rate of 34.3% (92/268). This is in fact lower than a report from south west Ethiopia and Nigeria [7-9]. WHO recommends conducting 3 stool examinations collected on separate days because of the intermittent shading of the oocysts [2]. This might be one possible reason for the lower prevalence of *Cryptosporidium spp* in this paper. For instance, a report from Malawi has detected an additional 13% of *Cryptosporidium parvum* after conducting the second stool examination [9]. In addition, it is also important to consider the reported seasonal variations in the prevalence of *Cryptosporidium spp* infections [10,11]. However, there are also papers from Ethiopia [12], Malaysia [13], India [14] and Iran [15] that have reported relatively lower prevalence of *Cryptosporidium spp* than the current prevalence. One possible reason for this could be that most of our study participants had complaints of diarrhea (90.3%) which may increase the detection yield of the parasites.

Similar to the findings with Certad et al., we have not seen gender difference in the prevalence of *Cryptosporidium spp* infection [16]. In addition, the role of CD4 cell counts seems to be insignificant on the prevalence of *Cryptosporidium spp* infection in this report. However, the effect of CD4 cells count on the prevalence of *Cryptosporidium spp* infection has been well documented and is biologically well explained [14,17-19]. In this study CD4 counts were not measured at the time of the stool specimen collection; instead, we have taken the most recent CD4 counts that were documented in the HIV care clinic of the hospital. In fact it might have happened that the CD4 counts of patients may in reality be higher (for patients who started treatment) or lower (for HAART naïve patients) than the collected data by the time of stool sample collection. The median recent CD4 counts among patients infected with *Cryptosporidium spp* was 166.0 (IQR 80.8, 266.0) where as it was 192.0 (IQR 112.8, 280.0) among uninfected patients.

A couple of articles reported that after initiation of treatment with HAART, it is possible to eradicate *Cryptosporidium spp* among immunocompromised patients [3,20]. In these two papers, the study patients were on second line treatment regimen consisting of protease inhibitors (PI) and were reported from high income countries. On the contrary none of our study patients were on PI. This could be a potential researchable area as to the effectiveness of PI and non-PI containing HAART for the eradication of *Cryptosporidium spp*. In the current study majority (85.8%) of patients who have started HAART were within their first year of their treatment. The median durations to clear off infection with *Cryptosporidium spp* as reported by the two papers above were 13.6 months [3] and 6 months [20]. The numbers of patients followed up were only twelve [3] and six [20]. The latter paper has noted the importance of response to HAART treatment for eradication of *Cryptosporidium spp*. This could indicate that in the current study the patients were not able to clear off the infection after one year of their medication with HAART. But it could also be that some of the patients were having transient infection [20] or relapse of infection as was pointed out by Carr et al. [3]. The other papers that reported reduction in prevalence of *Cryptosporidium spp* after starting HAART failed to report the association between the reduction and duration of treatment [6,21]. Another factor to look at is the level of adherence of patients to their treatment regimen. The effectiveness of HAART highly depends on how adherent patients are on their treatment [22,23] but low level of adherence to first line treatment is a common phenomenon in low income countries including Ethiopia [24,25].

Concerning the main clinical features, we have observed that vomiting, reported weight loss and chronic diarrhea were strongly associated with *Cryptosporidium spp* infection. In order to control for the side effects of HAART, we have also included treatment status in to the regression analysis. The fact that chronic diarrhea is more common among *Cryptosporidium spp* infected patients than patients with no diarrhea has been reported in other literature [5,10,16,26]. It has been documented that patients with *Cryptosporidium spp* infection can have weight loss of up to 10% when they have stool frequency as high as 10 per day [4,16]. Even though we failed to objectively verify the degree of weight loss, we observed that there is strong association between reported weight loss and infection with *Cryptosporidium spp.*

The prevalence of *I. belli* was low when compared with other studies conducted previously [12,14,27]. One possible explanation for this could be the use of cotrimoxazole prophylaxis which is effective to control infection with *I. belli*.

## Conclusion

In summary, there is still high burden of infection with *Cryptosporidium spp* among HIV infected individuals in southern Ethiopia. We recommend considering infection with *Cryptosporidium spp* in HIV infected people with chronic diarrhea, weight loss and vomiting for HAART naïve patients and/or for patients who are within the first year of starting treatment with HAART. It is also very important to note that patients with acute diarrhea can also be suffering from this infection as they could be in the early phase of the clinical manifestation of the disease.

## Competing interest

The authors declare that they have no competing interests.

## Authors’ contributions

MG designed the study, collected both the field and lab data, and involved in the analysis and manuscript preparation. The original research was the requirement for his MSc in biomedical sciences/parasitology; BP and TE were primary thesis advisors. TE also involved in the manuscript preparation, WT reanalyzed the data and involved in the preparation of the manuscript. All authors read and approved the final manuscript.

## Pre-publication history

The pre-publication history for this paper can be accessed here:

http://www.biomedcentral.com/1471-2334/14/100/prepub
